# Swab Testing to Optimize Pneumonia treatment with empiric Vancomycin (STOP-Vanc): study protocol for a randomized controlled trial

**DOI:** 10.21203/rs.3.rs-4365928/v1

**Published:** 2024-06-18

**Authors:** Jeffrey A. Freiberg, Justin K. Siemann, Edward T. Qian, Benjamin J. Ereshefsky, Cassandra Hennessy, Joanna L. Stollings, Taylor M. Rali, Frank E. Harrell, Cheryl L. Gatto, Todd W. Rice, George E. Nelson, Vanderbilt Center for Learning Healthcare

**Affiliations:** 1 Division of Infectious Diseases, Department of Medicine, Vanderbilt University Medical Center, Nashville, Tennessee, USA.; 2 Vanderbilt Institute for Infection, Immunology and Inflammation, Vanderbilt University Medical Center, Nashville, Tennessee, USA.; 3 Vanderbilt Institute for Clinical & Translational Research, Vanderbilt University Medical Center, Nashville, Tennessee, USA.; 4 Division of Allergy, Pulmonary, and Critical Care Medicine, Vanderbilt University Medical Center, Nashville, Tennessee, USA.; 5 Department of Anesthesiology, Vanderbilt University Medical Center, Nashville, Tennessee, USA.; 6 Department of Pharmaceutical Services, Vanderbilt University Medical Center, Nashville, Tennessee, USA.; 7 Department of Biostatistics, Vanderbilt University School of Medicine, Nashville, Tennessee, USA.; 8 Medical Intensive Care Unit, Vanderbilt University Medical Center, Nashville, Tennessee, USA.; 9 Department of Emergency Medicine, Vanderbilt University Medical Center, Nashville, Tennessee, USA.

**Keywords:** Methicillin-resistant *Staphylococcus* aureus, vancomycin, community-acquired pneumonia, MRSA nasal swab, PCR testing, antimicrobial stewardship, ICU

## Abstract

**Background:**

Vancomycin, an antibiotic with activity against Methicillin-resistant *Staphylococcus aureus* (MRSA), is frequently included in empiric treatment for community-acquired pneumonia (CAP) despite the fact that MRSA is rarely implicated in CAP. Conducting polymerase chain reaction (PCR) testing on nasal swabs to identify the presence of MRSA colonization has been proposed as an antimicrobial stewardship intervention to reduce the use of vancomycin. Observational studies have shown reductions in vancomycin use after implementation of MRSA colonization testing, and this approach has been adopted by CAP guidelines. However, the ability of this intervention to safely reduce vancomycin use has yet to be tested in a randomized controlled trial.

**Methods:**

STOP-Vanc is a pragmatic, prospective, single center, non-blinded randomized trial. Adult patients with suspicion for CAP who are receiving vancomycin and admitted to the Medical Intensive Care Unit at Vanderbilt University Medical Center will be screened for eligibility. Eligible patients will be enrolled and randomized in a 1:1 ratio to either receive MRSA nasal swab PCR testing in addition to usual care (intervention group), or usual care alone (control group). PCR testing results will be transmitted through the electronic health record to the treating clinicians. Primary providers of intervention group patients with negative swab results will also receive a page providing clinical guidance recommending discontinuation of vancomycin. The primary outcome will be vancomycin-free hours alive, defined as the number of hours alive and free of the use of vancomycin within the first seven days following trial enrollment estimated using a proportional odds ratio model. Secondary outcomes include 30-day all-cause mortality and time alive off vancomycin.

**Discussion:**

STOP-Vanc will provide the first randomized controlled trial data regarding the use of MRSA nasal swab PCR testing to guide antibiotic de-escalation. This study will provide important information regarding the effect of MRSA PCR testing and antimicrobial stewardship guidance on clinical outcomes in an intensive care unit setting.

**Trial registration:**

This trial was registered on ClinicalTrials.gov on February 22, 2024. (ClinicalTrials.gov identifier: NCT06272994)

## Introduction

### Background and rationale {6a}

Methicillin-resistant *Staphylococcus aureus* (MRSA) is a critical antimicrobial resistant threat responsible for greater than 300,000 inpatient infections and 15,000 deaths per year in the United States [1, 2]. Community-acquired pneumonia (CAP) is a major driver of hospital antibiotic use. Nationally, there are around 600,000 CAP-related hospital admissions annually. However, MRSA is an infrequent cause of CAP, accounting for less than 1% of cases [3]. Despite this, MRSA is a commonly feared cause of CAP, which leads to the frequent use of vancomycin, an anti-MRSA antibiotic, in empiric CAP treatment [4].

Inappropriate antibiotic use can lead to avoidable adverse drug events and costs, as well as drive antimicrobial resistance. Empiric vancomycin use in patients hospitalized for pneumonia has demonstrated increased mortality, acute kidney injury (AKI), and secondary infections [5]. Up to two-thirds of patients receiving high dose vancomycin develop AKI [6, 7]. Additionally, bone marrow suppression, linear IgA bullous dermatosis, anaphylaxis, and life-threatening hypersensitivity reactions can be seen with vancomycin use [8–11]. Use of vancomycin also increases healthcare costs, as it requires careful monitoring due to its narrow therapeutic range and high risk of toxicity [12]. Therefore, reducing inappropriate use of vancomycin in patients with CAP has the potential to improve patient outcomes while at the same time providing overall cost savings.

There are growing data to support the use of MRSA nasal swabs as a screening tool to guide de-escalation of vancomycin use in CAP. A 2018 meta-analysis found using nasal swabs for MRSA screening had an overall 96.5% negative predictive value (NPV) for pneumonia, which was increased to 98.1% among patients with CAP or healthcare-associated pneumonia [13]. Multiple retrospective studies along with prospective studies utilizing MRSA nasal swab-based de-escalation protocols have shown MRSA nasal swab use to be effective in decreasing vancomycin use, hospital length of stay, rate of AKI, and associated costs without having negative effects on patient outcomes [14–22]. The use of MRSA detection in nasal swabs is now consistent with guideline-based management of CAP [23]. However, all of the aforementioned studies are quasi-experimental analyses, and there are no randomized controlled studies of the use of MRSA nasal swab guided antibiotic de-escalation.

In particular, MRSA nasal swab PCR testing has not been adequately studied in the ICU setting. Since ICU patients have a higher severity of illness, there is a tendency towards broader antibiotic coverage and more frequent use of empiric vancomycin [24–27]. While this means a greater likelihood of unnecessary vancomycin use, it is also possible that there is greater hesitancy to discontinue vancomycin based on the results of the MRSA PCR testing alone. Retrospective studies looking at the use of nasal swab-based screening for MRSA colonization have found that the test maintains a high NPV in multiple ICU settings [28–32]. A prospective antibiotic de-escalation study found that an intervention based on negative MRSA nasal cultures decreased anti-MRSA antibiotic use in an ICU [22]. Therefore, studying the use of MRSA nasal swab PCR testing in an ICU setting where unnecessary vancomycin use is likely highest, but also the hesitancy to discontinue antibiotics is the greatest, is important to understand the potential benefit from this intervention.

### Objectives {7}

The primary aim of this study is to determine the effect of implementing MRSA nasal swab PCR testing and antimicrobial stewardship guidance via a pager alert on the use of vancomycin in cases of CAP. We hypothesize that implementation of this intervention will lead to appropriately reduced use of vancomycin. Furthermore, as secondary aims we anticipate a reduction in vancomycin-associated adverse events and an overall cost-savings, all without increasing mortality or hospital readmissions.

### Trial design {8}

This is a single center, pragmatic, randomized controlled trial examining whether the use of MRSA nasal swab PCR testing with reporting of negative PCR results directly to the primary provider via a pager alert leads to decreased vancomycin utilization for critically ill adults with CAP when compared with usual care. Eligible patients will be randomized in a 1:1 ratio between the intervention and usual care arms. Patients randomized to the intervention arm will have a MRSA PCR nasal swab collected and sent to the clinical laboratory. If the nasal swab does not detect MRSA, providers will receive a pager alert informing them of the negative result, recommending discontinuation of vancomycin if clinically appropriate, and directing providers to further clinical guidance. Patients randomized to usual care will not have a protocolized MRSA nasal swab ordered. If they receive a MRSA nasal swab as a part of usual care, a negative result will not trigger a pager alert, although it will be reported in the electronic health record (EHR). A pharmacist in the MICU will provide direct, in-the-moment education regarding vancomycin use in the setting of negative MRSA nasal swab PCR results, as well. The decisions regarding ordering or discontinuation of antibiotics will remain at the discretion of the treating provider. Data will be collected from the EHR to determine the effect of the assigned intervention on outcomes.

## Methods: Participants, interventions and outcomes

### Study setting {9}

This trial is being conducted at a single site with all patients being enrolled in the Vanderbilt University Medical Center (VUMC) Medical Intensive Care Unit (MICU), an intensive care unit in a tertiary-care academic hospital located in Nashville, Tennessee, USA.

### Eligibility criteria {10}

Inclusion criteria:

- Adult (age 18 years old or older) patients admitted to the VUMC MICU from the VUMC Emergency Department or from a hospital floor.
- Suspicion for pneumonia on admission (defined as either “respiratory infection” as the selected indication for antibiotics or an order for a respiratory culture i.e., sputum culture, tracheal aspirate culture, or bronchoalveolar lavage culture).
- No topical nasal decolonization during hospitalization prior to collection of MRSA nasal swab PCR.
- Patient ordered to receive vancomycin or a pharmacokinetics consult for continuing vancomycin dosing, no later than 24 hours following their physical admission to the MICU.

Exclusion criteria

- Hospital stay of longer than 48 hours prior to MICU admission.
- Known to be a prisoner.

### Who will take informed consent? {26a}

As a nasal swab is a routine, non-invasive, minimal risk test available in clinical practice, the intervention in this trial is solely to provide clinical guidance, and obtaining informed consent was suggested as being impractical. This trial was approved by the VUMC Institutional Review Board (IRB #231724) with a waiver of informed consent. Additional rationale is provided below in the section - Ethics approval and consent to participate.

### Additional consent provisions for collection and use of participant data and biological specimens {26b}

Not applicable.

## Interventions

### Explanation for the choice of comparators {6b}

Participants will be randomized to either receive treatment for CAP per usual care (control group) or to receive treatment for CAP and include a nasal swab with PCR testing to detect the presence of MRSA. Observational studies suggest that there may be a reduction in vancomycin use as the result of using MRSA nasal swab PCR testing to guide antimicrobial stewardship decisions [14–22], but this has not been demonstrated in a randomized controlled trial.

### Intervention description {11a}

Participants randomized to the intervention arm of the study will have a MRSA nasal swab PCR ordered on admission to the MICU, prior to the routine topical nasal decolonization that occurs on all patients admitted to the MICU. For participants enrolled in the control arm, no orders for PCR testing will be placed by the study team. Once the MRSA nasal swab PCR has been completed, the results will be available in the EHR. For the subjects assigned to the intervention group who have a negative MRSA nasal swab PCR result, providers will receive a pager alert which informs them of the negative result and recommends they discontinue vancomycin, if clinically appropriate. The MICU pharmacist will also provide direct education on the test results.

### Criteria for discontinuing or modifying allocated interventions {11b}

Participants retain autonomy to refuse MRSA nasal swab testing, as with any other clinical procedure. The decision to continue or discontinue vancomycin, or any other antibiotics, will not be dictated by the study, but instead is left solely to the discretion of the medical provider.

### Strategies to improve adherence to interventions {11c}

Nursing staff in the MICU will receive education on the purpose of the study and the need for collection of MRSA nasal swabs prior to routine topical nasal decolonization. In addition to the direct, instantaneous antimicrobial stewardship guidance to be delivered through a pager alert as outlined above, general education about the use of MRSA nasal swab PCR testing to guide de-escalation of empiric vancomycin will be provided by the MICU pharmacists when a patient with a negative MRSA nasal swab PCR is continued on vancomycin.

### Relevant concomitant care permitted or prohibited during the trial {11d}

Providers will be allowed to order MRSA nasal swab PCR testing independent of this trial.

### Provisions for post-trial care {30}

There are no plans for additional follow-up or post-trial care outside of routine clinical care for the purpose of this trial. Any complications incurred would be those expected as part of routine care, and there is no compensation for participants in the trial.

### Outcomes {12}

#### Primary Endpoint

##### Vancomycin-free Hours Alive:

The primary outcome is an ordinal 3-level patient status assessed on 168 consecutive hours, with the ordinal status levels being alive and not on vancomycin, alive and on vancomycin, or dead. The statistical model explicitly considers death as a negative outcome that is a terminal event (absorbing state in state transition model terminology). The underlying statistical parameter representing the treatment effect is an odds ratio (OR) for staying or transitioning to states given the previous day’s state. An OR greater than 1.0 represents higher odds of vancomycin or death and higher odds of death conditional on the previous day’s state (which can only be one of the first two of the three possible states). The OR is translated to clinical restatements of the analysis model as follows (p-values from these other estimands are identical to the p-value from the underlying OR). The ordinal longitudinal transition model directly provides transition probabilities, and the law of total probability is used to convert these to unconditional probabilities (state occupancy probabilities – SOPs) such as the probability of being alive and vancomycin-free on the 48^th^ hour. The SOPs are summed over hours to estimate mean time in state, which is used to provide the estimate of the treatment difference in the mean number of vancomycin-free hours alive, which will be the first estimate reported.

#### Secondary Endpoint(s)

There are two prespecified secondary outcomes for this trial.

##### 30-day all-cause mortality:

Defined as mortality within 30 days with date of study enrollment as day 0.

##### Time Alive off Vancomycin:

The number of hours out of the 168 hours (7 days) following enrollment in the trial that the patient is alive and not receiving vancomycin. Time receiving vancomycin will be defined as the difference from time of administration of the first dose of vancomycin to time of administration of the last dose of vancomycin. If the patient was discharged prior to study day 7 and received vancomycin within 24 hours of discharge then the total duration of vancomycin will be calculated based on the anticipated last day of vancomycin according to the medication reconciliation at the time of discharge or else by using study day 7 as an end point, whichever is sooner.

#### Exploratory Endpoint(s)

There are multiple prespecified exploratory outcomes for this trial, which are defined below:

##### Hospital length of stay:

The difference in days from date of presentation to VUMC to the date of discharge, inclusive of first and last dates.

##### ICU length of stay:

The difference in hours from time of admission to an ICU to transfer out of an ICU (summed if multiple separate transfers to an ICU during the same hospitalization).

##### Total duration of antibiotic exposure:

The difference in days from time of first dose of any oral or intravenous antibiotic to the last dose of antibiotics, inclusive of first and last dates. If the patient was discharged prior to study day 14 and received antibiotics within 24 hours of discharge then the total duration of antibiotics will be calculated based on the anticipated last day of antibiotics at the time of discharge or else by using study day 14 as an end point, whichever is sooner.

##### Total duration of intravenous antibiotic exposure:

The difference in days from time of first dose of any intravenous antibiotic to the last dose of intravenous antibiotics, inclusive of first and last dates. If the patient was discharged prior to study day 14 and had received intravenous antibiotics within 24 hours of discharge then the total duration of intravenous antibiotics will be calculated based on the anticipated last day of intravenous antibiotics at the time of discharge or else by using study day 14 as an end point, whichever is sooner.

##### Ventilator-free days:

The number of days out of the 14 days following enrollment in the trial that the patient is alive and not mechanically ventilated.

##### Total cost of vancomycin use:

The estimated cost of vancomycin administration as a composite of cost of drug, cost of associated laboratory testing, and cost of pharmacy services required to provide dosing.

##### AKI:

An increase in creatinine to ≥ 1.5 times the reference creatinine level or an increase by ≥ 0.3 mg/dL within 14 days of study enrollment [33]. Excludes patients who have end stage renal disease prior to study enrollment.

##### Receipt of any alternative non-vancomycin anti-MRSA pneumonia antibiotics:

Receipt of linezolid or ceftaroline during hospitalization (daptomycin excluded given inappropriate antibiotic selection for pneumonia).

##### In-hospital all-cause mortality:

Mortality at any point during the hospital stay prior to discharge.

##### Predictive value/concordance with final culture-based results:

Among patients with both a MRSA nasal swab PCR and a respiratory culture at any point from admission to 7 days after study enrollment, this outcome is defined as the percentage of patients with a positive MRSA nasal swab PCR who also had any respiratory culture during the first 7 days after study enrollment that was positive for MRSA (Positive Predictive Value) and the percentage of patients with a negative MRSA nasal swab PCR who also had any respiratory culture during the first 7 days after study enrollment that was negative for MRSA, regardless of whether another pathogen was detected or not (Negative Predictive Value).

#### Implementation Endpoints

##### Time to Result:

Time from collection of swab to results being available in EHR

#### Fidelity Endpoints

##### Fidelity of Obtaining Nasal Swab:

Percentage of patients randomized to intervention arm who have a MRSA nasal swab collected.

##### Fidelity of Running and Reporting PCR Results:

Percentage of patients who have a MRSA nasal swab collected who have PCR testing performed and results reported in the EHR.

##### Fidelity of Paging Alert to Team:

Percentage of patients in the intervention arm with a negative MRSA nasal swab PCR test result who have pager alerts sent to their primary provider.

### Participant timeline {13}

A participant timeline is shown in Table 1.

### Sample size {14}

The method used for sample size estimation is based on a traditional univariate proportional odds model. The actual primary analysis is based on a longitudinal proportional odds model that makes use of hourly patient status. For the purposes of this study, a 24-hour reduction in vancomycin use is considered clinically meaningful.

To overcome challenges with mortality, which is estimated at 22% within 8 days[34], the primary outcome, ‘vancomycin-free hours alive’, is defined as the number of hours alive and free of the use of vancomycin within the first seven days of study enrollment. This is not a quantity that is calculated on raw data because of deaths but is derived from the longitudinal model described below. The result is summarized as a treatment difference in mean vancomycin-free hours alive for specific covariate settings (p-values are the same for all covariate settings since treatment is not allowed to interact with covariates). The result will also be summarized by an OR for the hour-to-hour state transitions, from the proportional odds longitudinal model.

In those who do not die, it was estimated from preliminary data that the relative frequencies for all possible outcomes will be equal to 0.023, 0.008, 0.019, 0.027, 0.095, 0.125, 0.224, 0.262 (for 0 to 7 vancomycin-free days). Given this distribution, and an assumption of 22% mortality, an overall 24-hour increase in mean ‘vancomycin-free hours alive’ would be associated with a common OR for a univariate proportional odds logistic model of about 2.2. To detect this difference with 90% power would require about 106 subjects per study group.

### Recruitment {15}

Patients will be automatically enrolled and randomized either upon transfer to the MICU with an order for “pharmacy consult for vancomycin dosing” already in place if a respiratory culture has been ordered, or when the indication for vancomycin or for “pharmacy consult for vancomycin dosing” is “respiratory infection.” If an order for vancomycin or a “pharmacy consult for vancomycin dosing” order is placed (within 24 hours of admission to the unit) for a patient located in the MICU, they will also be automatically enrolled if a respiratory culture has been ordered, or when the indication for vancomycin or for “pharmacy consult for vancomycin dosing” is “respiratory infection.”.

## Assignment of interventions: allocation

### Sequence generation {16a}

A software application in the EHR will randomize patients using a simple 1:1 randomization strategy.

### Concealment mechanism {16b}

Randomization will be concealed until an intervention group is assigned.

### Implementation {16c}

When an order for vancomycin or a consult for “pharmacy to dose vancomycin” is placed for a patient located in the MICU, or a patient is transferred to the MICU with an order for “pharmacy to dose vancomycin” already present, a software application in the EHR will automatically assess a patient’s eligibility for this study based on whether they meet the inclusion criteria and assuming they do not meet any of the exclusion criteria. Eligible patients will be automatically enrolled in this study and randomized in a 1:1 fashion to either the control or the study group by a software application in the EHR.

## Assignment of interventions: Blinding

### Who will be blinded {17a}

Given that this study relies on using diagnostic testing to provide guidance to providers, it is not feasible to blind providers in this study. Likewise, patients will not be explicitly blinded as this would not be feasible if the patient’s providers must discuss their rationale for discontinuing or continuing antibiotics as part of informed, shared decision making.

### Procedure for unblinding if needed {17b}

Not applicable as the study is unblinded.

## Data collection and management

### Plans for assessment and collection of outcomes {18a}

Data used for this trial will be collected as part of routine clinical care. Data necessary to assess outcomes will be collected either automatically from the EHR or by manual chart review with entry into a HIPAA (Health Insurance Portability and Accountability Act) compliant Research Electronic Data Capture (REDCap) database [35, 36]. Data will ultimately be compiled into a password protected, deidentified database. Data to be collected include, but are not limited to, participant demographics, laboratory values, MRSA nasal swab PCR testing results, antimicrobial administration details, bacterial culture results, respiratory support requirements, creatinine levels, and 30-day mortality.

### Plans to promote participant retention and complete follow-up {18b}

With the exception of 30-day mortality, the majority of data necessary to calculate trial outcomes will be collected during the course of a participant’s index hospitalization, therefore we anticipate very low rates of missing data. There are no study follow-up visits nor need to promote participant retention based on this study design.

### Data management {19}

Patients will be followed for 30 days or until hospital discharge, whichever is longer. Protected health information (PHI) will be stored in a secure, HIPAA compliant, online REDCap database. Data will be entered by trained study team members. The database has internal data quality checks including restrictions on entry types and range checks for data values.

### Confidentiality {27}

A minimal amount of PHI will be collected. Only key study personnel will have access to the secure, HIPAA compliant, online REDCap database containing PHI. Once the data collection period is complete, a deidentified research database will be created for data analysis and will be stored on a password protected VUMC server indefinitely. After data analysis is complete, PHI will no longer be accessed.

### Plans for collection, laboratory evaluation and storage of biological specimens for genetic or molecular analysis in this trial/future use {33}

Not applicable - no human biological specimens will be collected for research purposes for current or future genetic or molecular analysis.

## Statistical methods

### Statistical methods for primary and secondary outcomes {20a}

The initial analysis will be descriptive in nature, summarizing information that characterizes the cohort and the outcomes. An inferential analysis to answer the primary study question will follow. Finally, the secondary endpoints will be compared between study groups.

#### Descriptive Analysis

To characterize the study sample, baseline demographic and clinical data will be described overall and by group. Categorical variables will be described using frequencies and proportions, and continuous variables will be described using medians and interquartile ranges. Missingness will be reported for each variable. Graphical summaries using box plots, violin plots, and/or histograms may be used to describe the data graphically. At a minimum, the following variables will be described at time of enrollment:

- Encounter ID (patients could be re-enrolled if they have multiple admissions)
- Age (years)
- Sex (male, female, unknown)
- Race (African American, Asian/Pacific Islander, Caucasian, Multiple, Native American, Other, Unknown)
- Ethnicity (Hispanic, Non-Hispanic, Unknown)
- Height (m)
- Weight (kg)
- BMI
- Elixhauser comorbidity index
- Pre-existing chronic kidney disease (CKD)
  - Baseline CKD
  - Reference Creatinine - Defined as the most recent creatinine obtained since admission but prior to the administration of the first dose of vancomycin. If no value exists, then the most immediate creatinine following the administration of vancomycin will be used.
- End stage renal disease – defined as receiving kidney replacement therapy on admission
- Immunocompromised/immunosuppressed
  - Human immunodeficiency virus positive
  - Solid organ transplant recipient
  - Stem cell transplant recipient
- Severity of illness
  - Sequential Organ Failure Assessment score
  - Level of supplemental oxygen and/or need for mechanical ventilation
- Diagnostic microbiological workup
  - Number of blood cultures, respiratory cultures, and other respiratory pathogen specific tests bulleted per person

All of the outcome variables overall and grouped by study arm will be described using the same approach as for the demographic data. Summary statistics and graphical representations may be displayed, and missingness will be reported for each variable.

No statistical comparisons between groups will be done for this descriptive analysis. Please note, showing descriptive statistics stratified by treatment groups that were randomized can be easily misinterpreted, as all apparent imbalances are by definition due to chance. Descriptive statistics of all-comers need to be emphasized in randomized studies.

#### Main Analysis

Regarding statistical analysis procedures, the statistical analysis plan supersedes the methods described in this protocol. Primary and secondary outcomes will be analyzed using an intention-to-treat analysis. The primary analysis will use a first-order Markov ordinal longitudinal proportional odds (PO) state transition model with three levels of patient status assessed hourly for 168 hours as described under *Primary Endpoint* above. This model places death as the worst outcome and does not need to code death numerically. A traditional approach of using vancomycin-free hours alive with death coded as –1 has interpretation problems (and does not handle missing data) and neither means (because of the arbitrariness of –1) nor medians (because of excessive ties in the data) are adequate summary statistics. The underlying transition model is a partial PO model; *partial* because the effect of time is allowed to vary with the ordinal state being predicted. Put another way, vancomycin may be used earlier, and deaths occur later, and it is important for the model not to have restrictions on how the various events unfold over time. The model is adjusted for baseline covariates. The within-patient correlation structure in the 3-level statuses is flexibly handled by a Markov process. “First order” means that each transition is conditional only on the previous day’s state and not statuses that are earlier. The partial PO model will assume PO for all other effects including treatment.

Letting the outcome variable on day t be denoted by Y(t) with levels 0, 1, 2 (alive & vancomycin-free, alive & on vancomycin, dead), and letting expit denote the inverse logit transform, the ordinal logistic model for day t may be written as follows, with y=1 or 2.


Pr(Y>=y|Y(t−1),X)=expit(a(y)+X*beta+t*tau*[y=2])

Here a(y) is the intercept corresponding to y, X represents baseline covariates, linear time effect, and a binary treatment indicator, beta are the main regression coefficients, and tau is a special effect of time t when y=2 (the partial PO effect). [z] denotes a 0–1 indicator function that is 1 if z is true, 0 otherwise.

A detailed case study using the proposed method, with code and results, may be found at https://hbiostat.org/rmsc/markov.html

The primary analysis will use R version 4.3.1 or later using the R VGAM package to fit the partial PO model and the Hmisc package to compute SOPs. Results will be displayed descriptively as 3-level stacked bar charts by treatment, covariate adjusted.

Likelihood ratio tests will be used for pivotal tests. These have better performance than Wald tests.

Secondary and exploratory outcomes will also be modeled using the flexible family of generalized linear models with similar covariate adjustment. Statistically significant differences in secondary and exploratory outcomes will be calculated using either the Wilcoxon-Mann-Whitney non-parametric test for differences, the Chi-squared test, or Cox proportional hazards model with consideration given to the competing risk of mortality, whenever appropriate. Fidelity to obtaining nasal swabs, running the rapid PCR, and delivering results will be evaluated. The potential for differential treatment effects based on fidelity to the expected process of care may be explored.

### Interim analyses {21b}

Not applicable - no interim analysis will be conducted.

### Methods for additional analyses (e.g. subgroup analyses) {20b}

To determine whether effects of treatment on the primary endpoint depends on any of the baseline characteristics the interaction between the baseline characteristics and treatment effect will be tested. This will be done by adding, one-at-a-time the potential interacting factors to the model detailed above. A detailed strategy for assessing differential treatment effects may be found at https://hbiostat.org/bbr/ancova.html#modeling-differential-treatment-effect. The prespecified potential interacting factors are as follows, Elixhauser comorbidity index, immunocompromised/immunosuppressed status, baseline CKD, and severity of illness.

### Methods in analysis to handle protocol non-adherence and any statistical methods to handle missing data {20c}

The analysis for the trial will use an intention-to-treat approach to answer the effectiveness question posed. That is, participants will be evaluated by treatment group as assigned regardless of what was delivered. All eligible participants will be included.

Missingness in the primary outcome is not expected. If there are missing covariates, cases will not be excluded; multiple imputation with predictive mean matching for missingness will be used in adjusting covariates. There may be missingness in secondary or implementation outcomes. The cohort for which the outcome is available will be described, along with the results of the model evaluating treatment effects in this cohort. All model results will be summarized with point estimates and 95% confidence intervals (CIs), which will be emphasized over p-values when reporting the results for secondary and implementation outcomes. No adjustments for multiplicity will be made.

Additionally, a descriptive analysis of missingness tendencies is warranted. For example, logistic regression will be used to predict the probability of missingness of an outcome measurement with predictors that include baseline variables and other outcomes that are never missing.

### Plans to give access to the full protocol, participant level-data and statistical code {31c}

The protocol and statistical analysis plan will be available with this published trial protocol manuscript. The deidentified dataset will be available after publication of the outcomes upon reasonable request.

## Oversight and monitoring

### Composition of the coordinating center and trial steering committee {5d}

A trial steering committee consisting of the principal investigator (PI), members of the Center for Learning Healthcare, members of the antimicrobial stewardship committee, MICU representatives, and biostatisticians meets monthly to oversee the trial. Conduct of the trial on a day-to-day basis is handled by the PI and study team members.

### Composition of the data monitoring committee, its role and reporting structure {21a}

As this is a pragmatic clinical trial that will only utilize diagnostic testing to provide guidance as part of routine clinical care and does not involve any specific treatment or procedural intervention, a data monitoring committee was deemed unnecessary.

### Adverse event reporting and harms {22}

Adverse events (AE) related to this study are not anticipated, as it only provides guidance in the context of routine clinical care. However, for the purpose of this study, an AE would entail any instance where vancomycin was discontinued based on the results of false negative PCR testing (defined for this study as a negative MRSA nasal swab in instances where a patient is subsequently determined to have a MRSA CAP as evidenced by growth of MRSA from a bacterial blood or respiratory culture collected within 48 hours of the MRSA nasal swab). In particular, any AE which results in or is associated with death, is life-threatening, prolongs hospitalization, or triggers a medical event or intervention to prevent one of these from occurring, will be considered a serious AE (SAE). If any AEs are identified, study personnel will monitor the safety of subjects and follow the AE until the event resolves or is explained.

To ensure proper and timely reporting of all AEs, there will be a clear communication plan for all study personnel to follow. Related or unexpected AEs will be recorded in the AE case report form (CRF) in the electronic database and reported to the PI within 5 days of occurrence. The PI will provide a report of all related or unexpected AEs that occur annually to the IRB as part of the annual review process. All related or unexpected SAEs will be reported to the PI within 72 hours of occurrence. The PI will, in turn, report all unexpected deaths, serious and treatment related adverse events, and serious and unexpected suspected adverse reactions to the IRB within 7 days after receipt of the report. A written report will be sent to the IRB within 15 calendar days.

### Frequency and plans for auditing trial conduct {23}

Not applicable - there are no plans for auditing trial conduct.

### Plans for communicating important protocol amendments to relevant parties (e.g. trial participants, ethical committees) {25}

Any protocol amendments will be approved by the IRB prior to implementation and will be communicated promptly to all of the key study personnel via email. Applicable protocol changes will be updated on ClinicalTrials.gov, as well.

### Dissemination plans {31a}

Results from this trial will be communicated through presentations at appropriate research meetings and submission for publication in peer-reviewed scientific journals in a timely fashion. Results will be released to ClinicalTrials.gov, and data resulting from this trial will be made available as required by federal data sharing policies. Results will be circulated to all study team members, and any publication of the study results will be approved by all investigators prior to publication.

## Discussion

STOP-Vanc will be the first randomized trial to study the use of PCR testing of nasal swabs to detect MRSA for the purposes of antimicrobial stewardship. However, there have been many observational studies that show a reduction in vancomycin use associated with the use of MRSA PCR testing, and the use of MRSA nasal swab testing has already been incorporated into the latest CAP guidelines (22). This makes a pragmatic clinical trial studying its use in an ICU all the more important. The decision to conduct this trial in an ICU was based on several factors. First, patients in the ICU are more likely to have severe CAP, and therefore administration of the correct empiric antibiotics is of the utmost importance. Presumably. this population is likely to have greater unnecessary vancomycin use and increased hesitancy to discontinue vancomycin, regardless of MRSA nasal swab PCR testing results. This potential bias towards continuing vancomycin in patients with a higher severity of illness may have been missed in some observational studies, as providers may not choose to order MRSA nasal swab PCR testing if they are not planning on changing their management based on the results. Therefore, by studying the use of this test in a setting likely to have a high degree of unnecessary vancomycin use along with hesitancy to discontinue antibiotics, this study is designed to maximize the ability to find an antimicrobial stewardship benefit from MRSA nasal swab PCR testing, if it exists.

An important consideration in the design of this study was how to identify patients with CAP. In practice, while there may be a suspicion for pneumonia at the time of antibiotic initiation, the actual diagnosis may not be made until later in a patient’s hospitalization. To study the benefit of early MRSA PCR testing on empiric vancomycin use, this trial was designed to prioritize prompt collection of nasal swabs for PCR testing. This study employs a strategy to quickly identify patients who are likely to or suspected to have CAP based solely on EHR elements. The presence of a respiratory culture will be used as a surrogate for suspicion for pneumonia. Alternatively, because cultures are not always ordered prior to the initiation of antibiotics, the indication for vancomycin administration will also be used as a surrogate. At VUMC, an indication for antibiotics must be selected at the time of ordering from a list of pre-specified options. Included on this list is “respiratory infection” as an indication. Any patients who otherwise meet the inclusion study criteria and have vancomycin ordered with this indication will be enrolled. This strategy was adopted as it was felt it would maximize the chances of promptly enrolling the target patient population. However, it is inevitable that this strategy will include some participants with a final diagnosis besides CAP. The number of such participants should be limited based on the enrollment strategy and should not impact the overall study results. These patients still represent the overall population of patients receiving empiric vancomycin, and although less studied, there are data to support a high NPV for MRSA PCR testing in infections outside of the respiratory tract [37–42]. Furthermore, patients with an initial suspicion for CAP but a different final diagnosis are part of the group that would be likely to receive this intervention in actual practice settings, making it important to ensure that those participants have no adverse outcomes, as well.

A potential limitation of this study is the fact that it is being conducted in a single ICU at a single center. While this may limit the generalizability of the results, this study would be difficult to standardize across multiple sites, at least as currently envisioned. The trial was carefully designed based on the specific patient and provider workflow at this institution along with the requirements of the specific EHR system in use to prioritize the early collection of nasal swabs for PCR testing, and therefore maximizing the potential benefit of this intervention. The specific details may be challenging to replicate exactly in a comparable study design elsewhere. However, if successful, this study could provide a blueprint for other institutions to adopt a similar approach tailored to their own institution’s practices and procedures.

In summary, STOP-Vanc is a single-center, unblinded, pragmatic randomized clinical trial that will compare the use of MRSA nasal swab PCR testing in patients admitted to an ICU. This trial will provide important and needed information about the potential for PCR testing coupled with antimicrobial stewardship guidance to reduce unnecessary vancomycin use.

## Figures and Tables

**Table 1:** Standard Protocol Items: Recommendations for Interventional Trials

	Study Period			
	Allocation and Enrollment	Post allocation		Final outcome assessment
TIMEPOINT	Transfer to MICU or order entry for vancomycin	7 days after enrollment	14 days after enrollment	Discharge or 30 days after enrollment
ENROLLMENT:				
EHR-based eligibility screening	✗			
Enrollment	✗			
Allocation	✗			
INTERVENTIONS:				
MRSA nasal swab PCR testing	✗			
No nasal swab PCR testing	✗			
ASSESSMENTS:				
Baseline variables	✗			
Antibiotic use	✗	✗	✗	
Adverse events		✗	✗	✗
Clinical Outcomes		✗	✗	✗

## Data Availability

The deidentified dataset will be available after publication of the trials results upon written request made to the corresponding author.

## Footnotes

Ethics approval and consent to participate {24} The use of a nasal swab to test for MRSA is minimal risk, as a nasal swab is not considered an invasive test and is currently used in routine care. Patients in the ICU receive twice daily nasal swabs for nasal decolonization while in the ICU as part of routine clinical practice. The risk to patients from the nasal swabs to detect MRSA is less than the risk from the routine nasal decolonization swabs as the swabs for MRSA detection do not contain any drugs. These routine nasal decolonization swabs are done as part of regular care without written consent from patients. Patients will have the same rights to refuse nasal MRSA swabs as their ability to refuse the routine nasal decolonization. In addition, this study does not make any decisions regarding the treatments offered to patients. Instead, it only provides information and antimicrobial stewardship guidance to patient’s providers who retain complete autonomy over what is the appropriate care for their patients. In addition to the minimal risk posed by the study, obtaining informed consent prior to enrollment in this study would not be feasible or practicable for multiple reasons:

1. The potential benefit of MRSA screening is dependent on early, rapid testing to generate results that are readily available to help guide the providers’ decisions. This means that the swab must be ordered immediately at the time that the ICU provider decides to start or continue antibiotics, and prior to routine decolonization that is performed in all patients admitted to the ICUs at Vanderbilt University Medical Center. This event typically occurs simultaneously with a patient’s arrival to the ICU. If the decision to obtain a MRSA nasal swab is delayed until written consent is acquired, it would be too late to study its effect.
2. The MRSA nasal swabs are only considered valid if they are collected prior to nasal decolonization. Since nasal decolonization with the use of nasal swabs occurs regularly twice a day in all ICU patients at VUMC, and most critically ill patients are temporarily cognitively impaired due to their critical illness upon entry to the ICU, it would not be possible to obtain written consent prior to decolonization in most cases.
3. The goal of the study is to test whether the use of a MRSA nasal swab would reduce the length of time that patients receive unnecessary antibiotics; thus, it must be done at the same time that the decision to start or continue antibiotics is made. Since the use of antibiotics in the ICU is an urgent intervention in many patients, it would be inappropriate and likely detrimental to delay the receipt of antibiotics in some of the most critically ill patients in order to get written consent for either the MRSA nasal swab or participation in the study.
4. The decision to initiate antibiotics is often made when patients are critically ill and unable to consent for themselves; therefore, it would often not be feasible to obtain consent from a patient or their representative at the time at which the MRSA nasal swab would need to be performed.
5. Consenting the participants may prompt either the clinical team or the participant to order (or ask for) the MRSA nasal swab in patients randomized to the control arm (and thus, randomized not to receive the nasal swab prior to decolonization). This would potentially lead the clinical team to order the MRSA nasal swab prior to decolonization in the control arm as well, which would not allow the study team to truly evaluate the role of an admission nasal swab in reducing vancomycin use. This contamination of the control arm would bias the study results to not being able to see the effect of the use of these pre-decolonization MRSA nasal swabs on vancomycin use.

For these reasons collectively, this study could not be done practicably without a waiver of consent, and it would be impractical to obtain written informed consent. Therefore, given that this study presents minimal risk, it would not adversely affect the welfare or privacy rights of the participant, and it would be impracticable to obtain consent, a waiver of informed consent was requested and approved for this study.

## Abbreviations

STOP-Vanc: Swab Testing to Optimize Pneumonia treatment with empiric Vancomycin
CAP: Community-acquired pneumonia
PCR: Polymerase chain reaction
MRSA: Methicillin-resistant *Staphylococcus aureus*
RCT: Randomized controlled trial
MICU: Medical Intensive Care Unit
VUMC: Vanderbilt University Medical Center
EHR: Electronic health record
ICU: Intensive care unit
AKI: Acute kidney injury
NPV: Negative predictive value
IRB: Institutional Review Board
OR: Odds ratio
SOPs: State occupancy probabilities
HIPAA: Health Insurance Portability and Accountability Act
PHI: Protected health information
CKD: Chronic kidney disease
PO: Proportional odds
AE: Adverse events
SAE: Serious adverse events
CRF: Case report form
PI: Principal investigator
